# The Expression and Localization of N-Myc Downstream-Regulated Gene 1 in Human Trophoblasts

**DOI:** 10.1371/journal.pone.0075473

**Published:** 2013-09-16

**Authors:** Xiao-Hua Shi, Jacob C. Larkin, Baosheng Chen, Yoel Sadovsky

**Affiliations:** 1 Magee-Womens Research Institute, Department of Obstetrics, Gynecology and Reproductive Sciences, University of Pittsburgh, Pittsburgh, Pennsylvania, United States of America; 2 Department of Obstetrics and Gynecology, Washington University, St. Louis, Missouri, United States of America; 3 Department of Microbiology and Molecular Genetics, University of Pittsburgh, Pittsburgh, Pennsylvania, United States of America; National Taiwan University, Taiwan

## Abstract

The protein N-Myc downstream-regulated gene 1 (NDRG1) is implicated in the regulation of cell proliferation, differentiation, and cellular stress response. NDRG1 is expressed in primary human trophoblasts, where it promotes cell viability and resistance to hypoxic injury. The mechanism of action of NDRG1 remains unknown. To gain further insight into the intracellular action of NDRG1, we analyzed the expression pattern and cellular localization of endogenous NDRG1 and transfected Myc-tagged NDRG1 in human trophoblasts exposed to diverse injuries. In standard conditions, NDRG1 was diffusely expressed in the cytoplasm at a low level. Hypoxia or the hypoxia mimetic cobalt chloride, but not serum deprivation, ultraviolet (UV) light, or ionizing radiation, induced the expression of NDRG1 in human trophoblasts and the redistribution of NDRG1 into the nucleus and cytoplasmic membranes associated with the endoplasmic reticulum (ER) and microtubules. Mutation of the phosphopantetheine attachment site (PPAS) within NDRG1 abrogated this pattern of redistribution. Our results shed new light on the impact of cell injury on NDRG1 expression patterns, and suggest that the PPAS domain plays a key role in NDRG1’s subcellular distribution.

## Introduction

NDRG1 (also called RTP, DRG1, CAP43, RIT42, TDD5, NDR1, and PROXY1) is a 394-amino acid protein that is implicated in cell differentiation, stress, and hormonal response [1-8]. Notwithstanding the ubiquitous expression of NDRG1 in most cell types and its upregulation in numerous types of cancer, a definitive analysis of NDRG1’s function *in vivo* pointed to a role limited to myelin sheath maintenance and regeneration. *Ndrg1* knockout mice exhibit peripheral neuropathy, hind limb weakness, and leg muscle atrophy at age 3 months [9,10]. The expression of *NDRG1* in murine Schwann cells is enhanced during regeneration after sciatic nerve injury [11]. These findings are attributed to a stop codon non-sense mutation (R148X) [12] or to an exon-9-skipping mutation (IVS8-1G>A, S181-K198) [13] found in humans with hereditary motor and sensory neuropathy-Lom (HMSNL, also known as Charcot-Marie-Tooth type 4D disease). This disease is characterized by Schwann cell demyelination and concomitant early axonal impairment affecting both motor and sense peripheral nerves, and resulting in a loss of limb muscle function and touch sensation in adulthood [12,13]. . In addition, NDRG1-null mice exhibit impaired mast cell differentiation and degranulation [8].

Using cultured primary human trophoblasts (PHT), we previously found that NDRG1 plays a pivotal role in the response of human placental trophoblasts to hypoxia, a common placental injury during pregnancy that is associated with impaired fetal growth [14,15]. . We found that hypoxia dramatically increases NDRG1 expression in PHT cells. Moreover, using overexpression and knock down approaches, we showed that NDRG1 enhances trophoblast differentiation and diminishes hypoxia-induced apoptosis [16]. Consistent with a role in placental adaptation to injury, enhanced expression of NDRG1 is associated with preeclampsia and fetal growth restriction [17].

Diverse chemical and cellular signals stimulate the expression of NDRG1, including reducing agents such as tunicamycin [18], metals (cobalt, nickel, calcium, and iron chelators) [19,20], nitric oxide [21], vitamin D [22], vitamin C [23], retinoids [7], androgens and estrogens [24-27], and DNA-damaging compounds (actinomycin D, doxorubicin, geldanamycin) [28-30].

While vital to cell function, the mechanism of action of NDRG1 remains unknown. NDRG1 is a member of the Ndr family of proteins [31]. All four members of this family harbor a conserved α/β hydrolase fold, yet lack its catalytic motif, rendering Ndr members devoid of hydrolase activity [32]. Moreover, the subcellular localization of NDRG1 is not uniform across cell types. NDRG1 can be found in the cytoplasmic membrane, desmosome and adherent junctions, mitochondria, vacuoles, intermediate and microfilament bundles, and cell nuclei and nucleoli [2,33-37].

To further our understanding of NDRG1’s function we investigated the impact of diverse cellular insults on the expression and subcellular distribution of NDRG1 in PHT cells and trophoblast lines. Unlike with hypoxia, we found that several other cellular insults had an insignificant effect on NDRG1 expression. Moreover, hypoxia caused a marked redistribution in NDRG1’s subcellular expression pattern, and this effect was dependent upon an intact phosphopantetheine attachment site (PPAS) motif within the α/β hydrolase fold of NDRG1.

## Materials and Methods

### Cell lines and culture

BeWo, JEG-3, CHO, and NIH3T3 lines were purchased from ATCC (Manassas, VA) and cultured as we previously detailed [38-41]. We also previously characterized and detailed the culture conditions of the uterine myocytes cell line SHM [42,43]. PHT cells were purified from term human placentas obtained by the Obstetrical Specimen Procurement Unit from women after a healthy pregnancy, labor, and delivery at Magee-Womens Hospital of the University of Pittsburgh Medical Center. The placental tissue samples were collected under an approved exempt protocol by the Institutional Review Board of the University of Pittsburgh. Patients provided written consent for the use of de-identified, discarded tissues for research upon admittance to the Hospital. Cells were isolated using the trypsin-deoxyribonuclease-dispase/Percoll method as described by Kliman et al. [44], with previously published modifications [45]. PHT culture density was 350,000 cells/cm^2^. Cells were maintained in DMEM (Sigma-Aldrich Corp., St. Louis, MO) supplemented with 10% FBS (HyClone, Logan, UT) and antibiotics. The cells were maintained in standard conditions (5% CO_2_ and 95% atmosphere, 37°C) before being exposed to serum-free starvation, CoCl_2_ (50-200 µM), or hypoxia (<1% O_2_) as previously described [39].

UV light irradiation of cells was carried out using a UV Crosslinker XL-1000 equipped with 254-nm tubes (Spectronics Corporation, Westbury, NY) at the energy of 6 mJ/cm^2^. For exposure to ionizing irradiation, PHT cells were irradiated using a Varian CLINAC 600C (Varian Medical Systems, Palo Alto, CA) with a 6-MV photon beam and dose rate of 250 cGy/min. Maximum radiation depth was 1.5 cm.

To determine protein decay, cells were exposed to the protein synthesis inhibitor cycloheximide (10 µg/ml, Sigma, St. Louis, MO) for 3, 6, 12, and 24 h at 48 h after transfection with plasmids expressing either myc-tagged NDRG1 wild type or myc-tagged PPAS deleted mutants. Cellular proteins were extracted, measured and processed for western blot as described below.

### Plasmid construction and transfection

A DNA vector encoding human NDRG1 was provided by Dr. Toshiyuki Miyata (National Cerebral and Cardiovascular Center, Japan). To generate plasmid constructs expressing Myc-tagged wild type or mutated NDRG1, the corresponding cDNA fragments were PCR-amplified and inserted into the NheI/BamHI site in pcDNA3.1/MycHisA(-) (Invitrogen, Carlsbad, CA). All mutant clones were confirmed by restriction digestion and DNA sequencing. Cells were transfected with 7.5 mM polyethylenimine as previously described [39].

### Isolation of RNA and quantitative real-time PCR

Total RNA extraction, RNA quality assessment, and reverse transcription were performed as we previously described [46]. Synthesized cDNA was diluted 1:5 in DNase- and RNase-free H_2_O (Invitrogen), and 3 µl of diluted cDNA per 10 µl of reaction mixture in a 384-well plate were used in duplicates of each sample for RT-qPCR, carried out in Geneamp 7900 using SYBR Green PCR master mix (Applied Biosystems, Forster City, CA). PCR primers (Integrated DNA Technologies, Coralville, Iowa) were validated using Primer Bank (Boston, MA). Human YWHAZ served as an internal control. Amplification specificity was routinely confirmed by dissociation curves. The results were calculated by the ΔΔC_t_ method [47] to determine the relative gene expression.

### Nucleus fractionation and Western blot analysis

The cell monolayer was washed, scraped in PBS, and centrifuged at 2,000 g for 5 min. The pellet was resuspended x5 v/v with buffer A (10 mM HEPES, 1.5 mM MgCl_2_, 10 mM KCl, 0.5 mM dithiothreitol (DTT) containing the protease inhibitors (1 mM PMSF, 10 µM leupeptin, 0.1 µM aprotinin, and 1 µM Pepstatin A) incubated on ice for 10 min, then centrifuged at 12,000 g for 15 sec at 4°C. The cytosolic supernatant was collected separately, and the nuclei-containing pellet was resuspended x3 v/v of buffer C (20 mM HEPES, 1.5 mM MgCl_2_, 420 mM NaCl, 0.2 mM EDTA, 0.5 mM DTT) containing protease inhibitors, as above, and incubated on ice for 1 h, followed by a vigorous vortex for 30 sec, centrifugation at 12,000 g for 2 min at 4°C, and collection of the nuclear extract-containing supernatant. Total cellular proteins were extracted by incubation of the cells in 50 mM Tris-buffered saline (TBS) containing 1% TX100, pH 7.4, on ice for 15 min. Cell debris was removed by centrifugation at 12,000 g for 15 min.

Total protein concentration was measured with a BCA protein assay kit (Thermo Scientific, Waltham, MA), using a microplate reader (VersaMax, Molecular Devices, Sunnyvale, CA). Extracted proteins from whole cell lysate or from subcellular compartments were separated by SDS-PAGE electrophoresis in 10% acrylamide/Bis gel (Bio-Rad, Hercules, CA) and then transferred to polyvinylidene difluoride membranes (Bio-Rad) at 25 V overnight. After blocking with 50mM Tris-buffered saline with 0.1% Tween-20 (TBST) containing 5% non-fat milk, the membranes were incubated overnight with rabbit polyclonal anti-NDRG1 antibody (final concentration 0.5 µg/ml, Invitrogen) or mouse monoclonal anti-Myc antibody (1 µg/ml, Applied Biological Materials, Richmond, BC) at 4°C. HIF-1α was detected using a mouse monoclonal anti HIF-1α antibody (2 µg/ml, BD Biosciences, San Jose, CA). Tubulin was detected using mouse anti-tubulin monoclonal antibodies (1 µg/ml, Calbiochem, San Diego, CA). After washing with TBST, the membranes were incubated with horseradish peroxidase-conjugated goat anti-rabbit or goat anti-mouse immunoglobulin G (0.15 µg/ml, Jackson ImmunoResearch, West Grove, PA) for 2 h at room temperature. Chemiluminescent signal was detected by enhanced SuperSignal West Dura (Thermo Scientific) for endogenous low level NDRG1 in cells cultured standard conditions, or by SuperSignal West Pico for all other conditions, using UVP Biospectrum 310 (UVP, Upland, CA).

### Immunofluorescence staining

Cells were plated on cover glass in a 12-well plate as described earlier, then fixed with 4% paraformaldehyde for 15 min, except for desmosome staining, for which the cells were fixed with ice-cold methanol at -20°C for 20 min. After rinsing with PBS, the cells were permeabilized and blocked with 0.1% Triton X-100 in 50 mM Tris-HCl (pH 7.4) containing 150 mM NaCl, 0.2 mg/mL NaN_3_, and 1 mg/ml BSA at room temperature for 1 h. The cells were stained with rabbit polyclonal anti-NDRG1 antibody (1 µg/ml) or mouse monoclonal anti–Myc tag antibody (1 µg/ml, Applied Biological Materials) for 4 h at room temperature. Cytoplasm membranes and desmosomes were stained by rabbit monoclonal anti-E-cadherin antibody (0.25 µg/ml, Cell Signaling, Danvers, MA) and mouse monoclonal anti-desmosome antibody (20 µg/ml, Sigma), respectively. Chicken polyclonal anti-calreticulin (0.5 µg/ml, Abcam, Cambridge, MA) and mouse anti-tubulin monoclonal antibodies (0.25 µg/ml, Calbiochem) were used to stain ER and microtubules. Peroxisomes were stained with rabbit polyclonal PMP70 antibody (2 µg/ml, Abcam). After five washes with PBS, primary antibodies were detected by 2 h incubation with the corresponding fluorophore-conjugated secondary antibodies (Alexa Fluor 488 or Alex Fluor 555). Actin filaments were stained with FITC-phalloidin (50 nM) at room temperature for 20 min. Nuclei were stained with Hoechst 33342 (5 µg/ml) or SYTOX Green (5 µM) for 5 min at room temperature, after incubation with the secondary antibody. Mitochondria and lysosomes were traced in live cells by incubation with MitoTracker Red (100 nM) and LysoTracker Red (50 nM), respectively, at 37°C for 30 min immediately before fixation. All antibodies and fluorescent dyes were purchased from Invitrogen unless otherwise stated. Images were captured using a 60X lens plus 1-2X digital zoom using the Nikon fluorescent microscope Eclipse Ti, equipped with Nikon’s A1 laser confocal system, and analyzed by NIS-Elements (Nikon Instruments, Melville, NY).

### Statistics

All experiments were repeated at least three times. Data are presented as means ± S.D, where relevant. Statistical analysis was performed using Student’s t-test based on log_2_ transformed fold change of mRNA expression. A p-value < 0.05 was considered significant.

## Results

### Hypoxia and CoCl_2_, but not non-hypoxic insults, enhance NDRG1 expression in PHT cells

**Figure 1:**
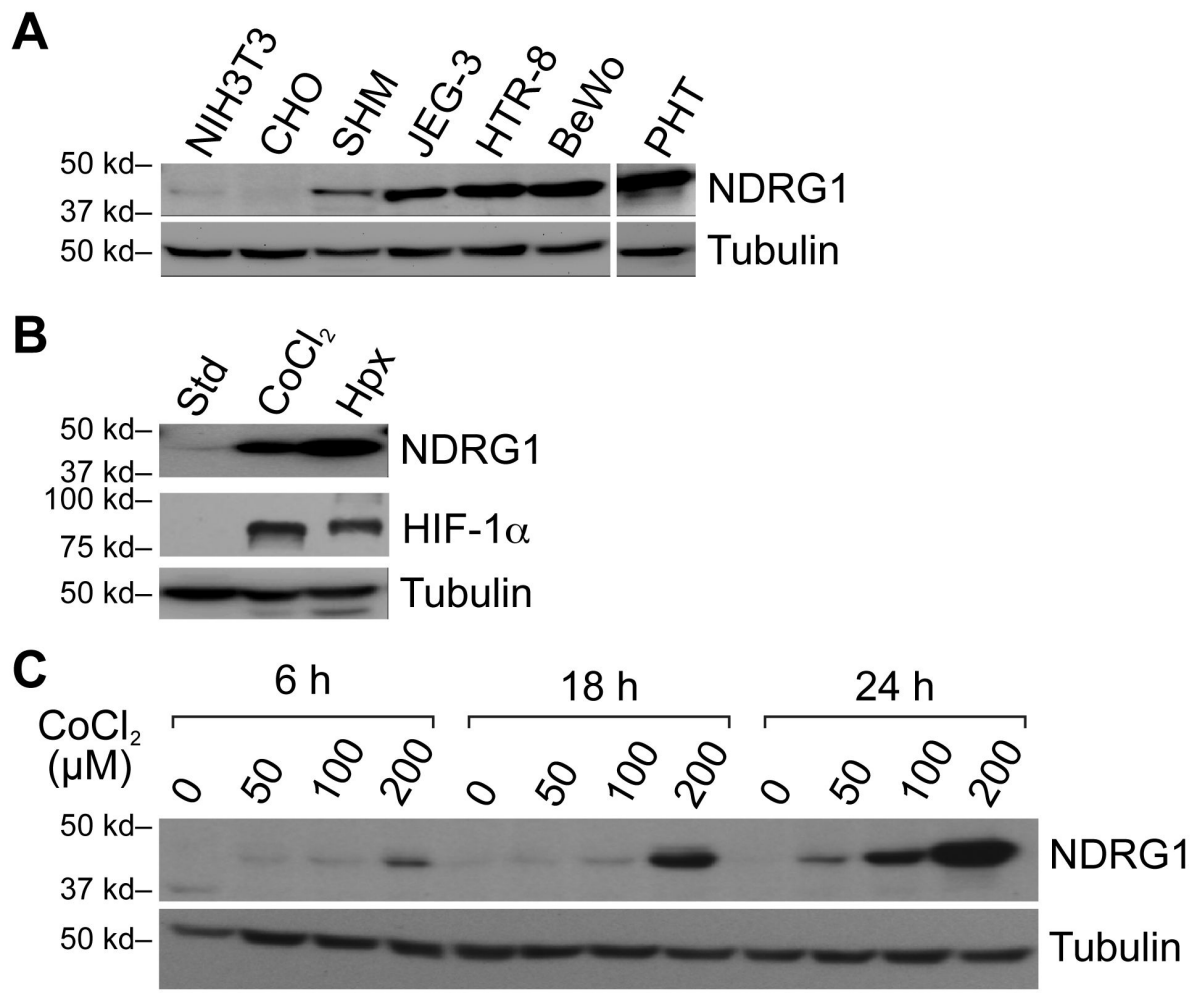
NDRG1 expression in trophoblast cell lines in response to hypoxia or CoCl_2_. NDRG1 (upper panels) and tubulin (loading control, lower panels) were detected using Western immunoblot. (A) NDRG1 expression in trophoblasts and trophoblast-derived choriocarcinoma cell lines, as well as in other cell types under standard conditions. (B) NDRG1 expression in JEG-3 cells cultured in standard conditions, 24 h of hypoxia (<1% O_2_) or the hypoxia mimetic CoCl_2_ (200 µM). The expression of HIF-1α (middle panel) confirmed the effect of CoCl_2_ or hypoxia. (C) A time course and concentration-dependent stimulation of NDRG1 expression in JEG-3 cells exposed to the hypoxia mimetic agent CoCl_2_. Data are representative of at least three independent experiments.

We previously showed that NDRG1 is expressed in human placental tissue as well as in cultured term PHT cells [16,48]. Taking advantage of their wide use in trophoblast biology as well as their accessibility to molecular manipulations, we used trophoblast lines, alongside PHT cells, to assess the impact of diverse cellular injuries on NDRG1 expression. We initially determined that the expression of NDRG1 protein in the trophoblast cell lines JEG-3, BeWo, and HTR-8/SVneo was markedly higher than other non-placental cell types, such as the myometrial line SHM, the ovarian line CHO, and NIH3T3 fibroblasts (Figure 1A). Hypoxia, as well as the hypoxia mimetic CoCl_2_, stimulated NDRG1 expression in JEG-3 cells (Figure 1B-C) in a manner similar to that found when using PHT cells [16].

**Figure 2:**
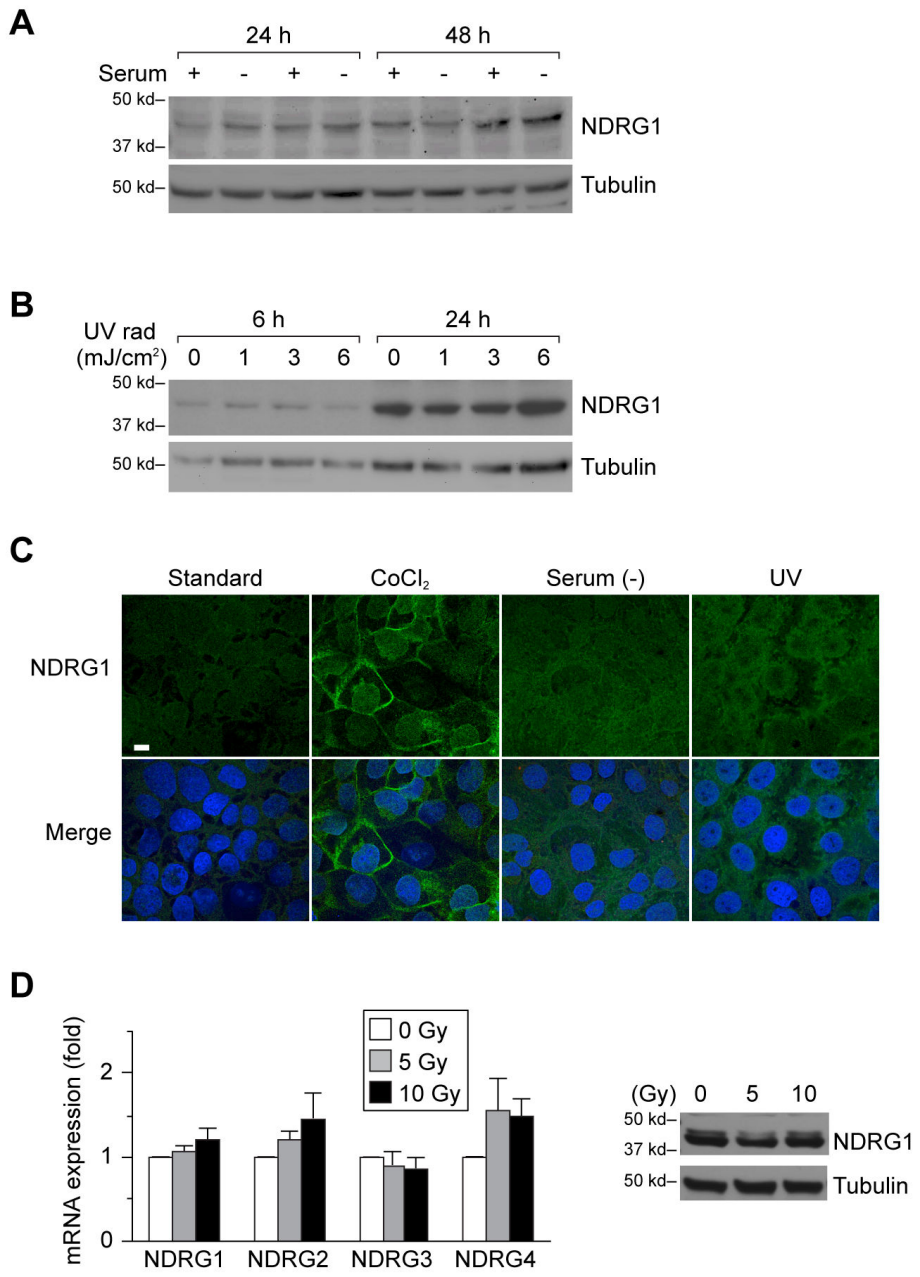
The impact of non-hypoxic cell injury on NDRG1 expression in trophoblast lines. (A) BeWo cells were incubated for either 24 or 48 h in standard, serum-containing medium or in serum-free medium. NDRG1 was detected by Western immunoblot (upper panel), and tubulin (lower panel) used for loading control. (B) JEG-3 cells were exposed to UV irradiation at the energy dosage indicated, and harvested after 6 or 24 h after the brief (1-3 seconds) pulse of UV irradiation. Analysis was performed as described in panel A. (C) Immunofluorescent staining of NDRG1 in JEG-3 cells 24 h after exposure to CoCl_2_ (200 µM), serum-free medium, or UV irradiation (6 mJ/cm^2^). Panels are NDRG1 alone, or channel merge of NDRG1 (green) and Hoechst 33342 (blue), used for nuclear staining. Bar = 20 µm(D) The expression of NDRG members in PHT cells 24 h after exposure to ionizing radiation at the energy dose shown. Left panel shows RT-qPCR analysis, performed as described in Materials and Methods. None of the differences were significant. Right panel depicts NDRG1 expression, detected as in panel A, in cells exposed to ionizing radiation at the energy dosage shown. Analysis was performed as described in panel A. Data are representative of at least three independent experiments.

To assess the influence of non-hypoxic cellular stress on NDRG1 expression, we cultured BeWo cells in the presence or absence of serum, and found that this had an insignificant effect on NDRG1 expression (Figure 2A). Although UV irradiation causes cell damage [49] (data not shown), we observed no effect of UV irradiation on NDRG1 protein levels in JEG-3 cells (Figure 2B) or in BeWo or HTR-8/SVneo cells (data not shown). These results were confirmed using immunofluorescent staining in JEG-3 cells (Figure 2C). Whereas ionizing irradiation promotes trophoblast cell death (manuscript in preparation), it had no effect on NDRG1 mRNA or protein expression (Figure 2D). Similarly, there was no effect on mRNA expression of other NDRG family members in PHT cells (Figure 2D). Together, these data indicate that hypoxia, but not other tested cellular stressors, enhances trophoblastic NDRG1 expression.

### Hypoxia causes subcellular redistribution of NDRG1 expression

**Figure 3:**
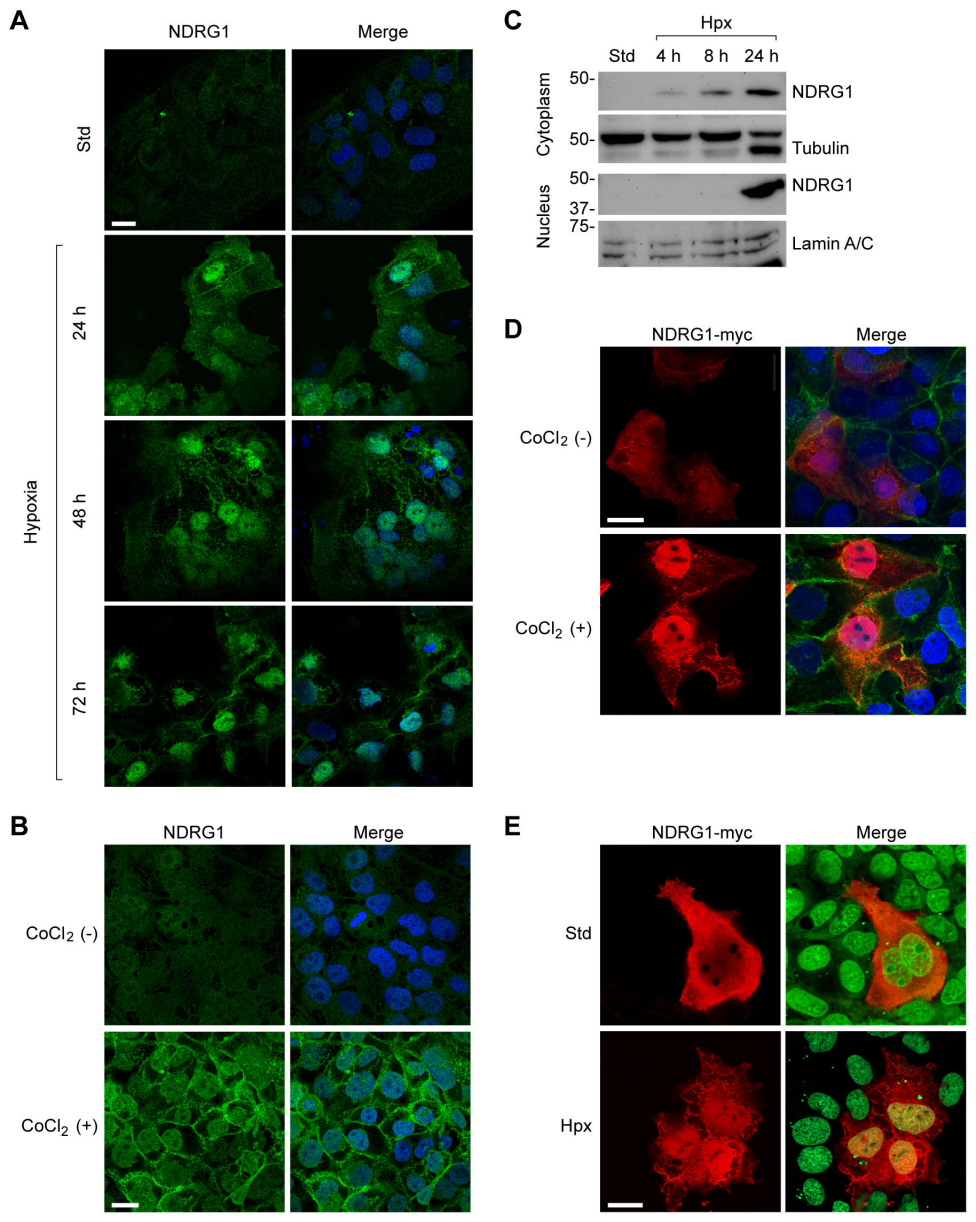
The sub-cellular localization of NDRG1 in response to hypoxic injury. Wild type or myc-tagged NDRG1, as well as cell nuclei, were detected as described in Materials and Methods. (A) The impact of hypoxia on NDRG1 expression level and localization in PHT cells exposed to hypoxia at the time period indicated. NDRG1 (green) is shown in the left panels, and a merged channel of NDRG1 and nuclei (blue) is shown in the right panels. (B) JEG-3 cells were exposed to CoCl_2_ (200 µM) for 24 h, and data analyzed as in (A). (C) A Western immunoblot of NDRG1 expression in the cytoplasmic (upper panel) or nuclear (lower panel) fraction of JEG-3 cells cultured in standard or hypoxic conditions for the period of time indicated. Tubulin and lamin A/C were used as markers of the cytoplasm and nucleus, respectively. (D) The impact of CoCl_2_ (200 µM for 24 h) on the localization of myc-tagged NDRG1 in JEG-3 cells. Myc-tagged NDRG1 (red), E-cadherin (green), and nuclei (blue) are shown. (E) The impact of hypoxia (24 h, <1% O_2_) on the localization of myc-tagged NDRG1 in BeWo cells. Myc-tagged NDRG1 (red) and nuclei (green) are shown. Data are representative of at least three independent experiments. Bar = 20 µm in all panels.

**Figure 4:**
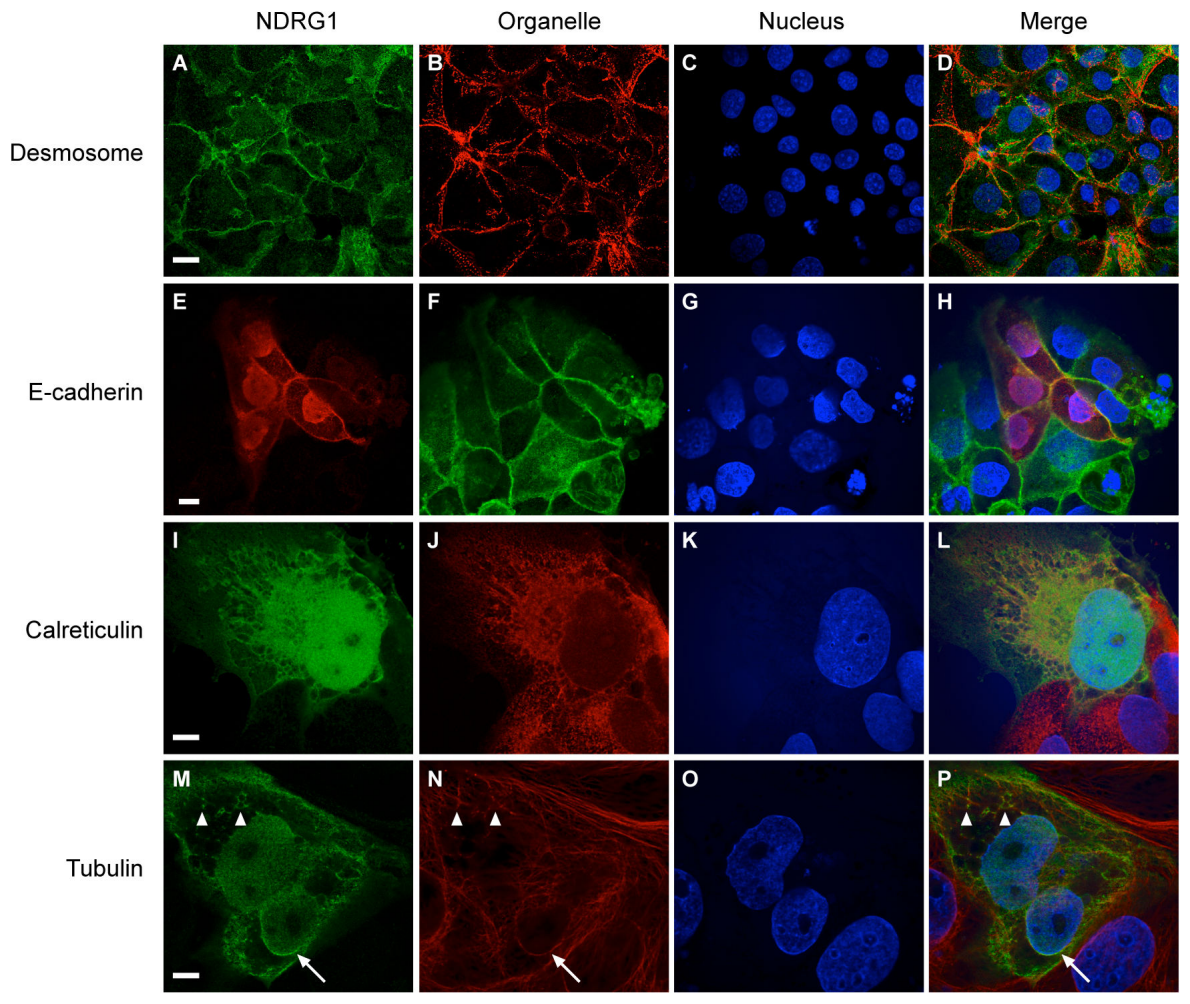
The expression of NDRG1 in cytoplasmic membranes or membrane-associated structures in hypoxic (24 h) trophoblastic lines. The nuclei (blue), in all panels, were detected using Hoechst 33342 and each organelle was detected using a specific antibody as described in Materials and Methods. (A-D) NDRG1 (green) co-localizes with desmosomes (red) in JEG-3 cells. (E-H) Myc-tagged NDRG1 (red) co-localizes with E-Cadherin (green) in BeWo cells. (I-L) Myc-tagged NDRG1 (green) co-localizes with calreticulin (red) in BeWo cells. (M-P) Myc-tagged NDRG1 (green) co-localizes with tubulin (red) in BeWo cells. Arrow points to perinuclear NDRG1 and tubulin signals. Arrowheads point to peripheral microtubules. Data are representative of at least three independent experiments. Bar = 20 µm in panels A-H, and Bar = 10 µm in panels I-P.

To test whether hypoxia impacts the subcellular localization of NDRG1, we cultured PHT cells under hypoxia for 24–72 h. Whereas, under standard conditions, NDRG1 was diffusely expressed in the cytoplasm and nucleus, hypoxia caused the redistribution of NDRG1 to the cell membrane and nucleus of PHT cells while sparing the nucleoli (Figure 3A). We observed a similar effect using JEG-3 cells exposed to CoCl_2_ (Figure 3B), and this finding was corroborated by cell fractionation and Western immunoblotting (Figure 3C). Overexpression of a Myc-tagged NDRG1 in CoCl_2_-exposed JEG-3 (Figure 3D) or hypoxic BeWo cells (Figure 3E) confirmed these results, showing a clear transition of NDRG1 from diffuse cytoplasmic to nuclear and membrane localization. As shown in Figure 4, hypoxia also enhanced NDRG1 expression at cytoplasmic membranes, where NDRG1 partly co-localized with desmosomes (Figure 4A-D) and with E-cadherin-positive membranes (Figure 4E-H), calreticulin-positive ER, (Figure 4I-L), and cytoplasmic and perinuclear tubulin (Figure 4M-P). In contrast, we found no evidence for localization of NDRG1 to trophoblastic mitochondria, lysosomes, Golgi, peroxisomes, or microfilaments (Figure S1).

### PPAS motif and the cap-like domain within the α/β hydrolase fold of NDRG1 are essential for protein localization and stability

**Figure 5:**
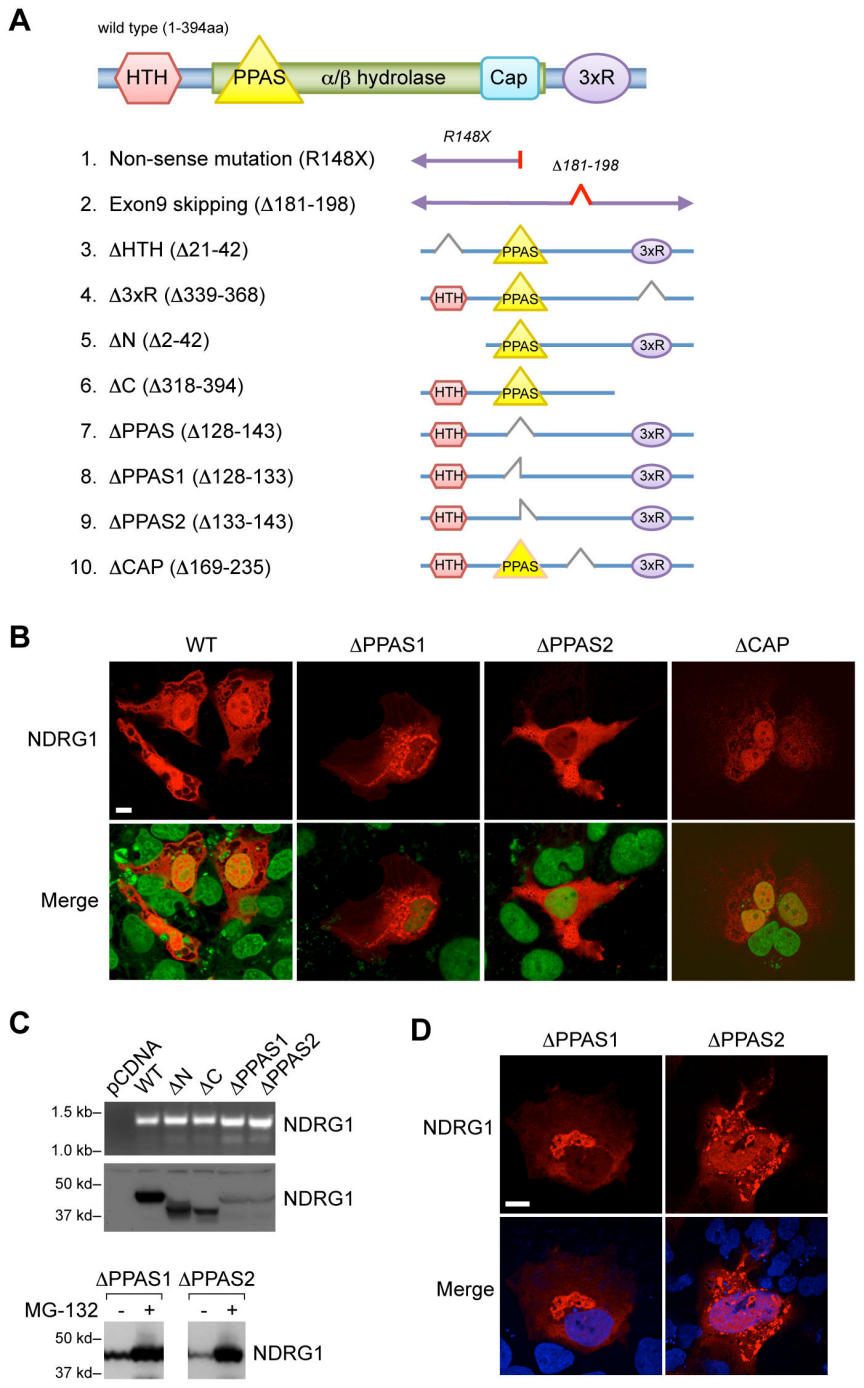
The expression and nuclear localization of NDRG1 deletion mutants. (A) Schematic representation of human NDRG1 protein structure, depicting protein domains, including those mutated in NDRG1-related human diseases, as well as NDRG1 deletion mutants generated in this study. (B) The subcellular localization of PPAS-deleted NDRG1 mutants in BeWo cells, cultured in hypoxia (24 h, <1% O_2_). Myc-tagged NDRG1 wild type and ΔCAP but not ΔPPAS (red, upper panels) were co-localized with nuclei (SYTOX Green, lower panels). Bar = 20 µm. (C) Upper panel: The expression of myc-tagged NDRG1 wild type or mutants (mRNA, upper gel; protein, lower gel) in BeWo cells. Lower panels: the effect of the proteasome inhibitor MG-132 (5 µM, 16 h) on the expression of myc-tagged NDRG1 mutant in PPAS domain in BeWo cells, detected using Western immunoblot. (D) The effect of the proteasome inhibitor MG-132 (5 µM, 16 h) on the subcellular localization of transfected mutant PPAS domain of NDRG1 (red) in hypoxic BeWo cells. Bar = 10 µm. Data are representative of at least three independent experiments.

The conserved α/β hydrolase fold (Figure 5A) in Ndr family members lacks a hydrolytic motif, rendering the protein devoid of hydrolase activity [32,50,51]. As NDRG1 intensely localizes to the nucleus upon exposure to hypoxia, we sought to identify domains within NDRG1 that direct this subcellular redistribution. *In silico* analysis (Network Protein Sequence Analysis, Lyon, France) pointed to a potential nuclear localization motif, helix-turn-helix (HTH) [52-54], at the N-terminal of NDRG1.

Other sequences within NDRG1 that are not conserved in other Ndr members include a unique three repeats (3xR) of 10-amino acids at the protein’s C-terminal, which is known to bind nickel [55], and the PPAS [56], located within the α/β hydrolase fold at residues 128-143. In acyl carrier proteins (ACP) of multi-enzyme complexes, such as fatty acid synthase, the PPAS sequence provides an attachment site to a prosthetic group that serves as a “swing arm” for easy access to activated fatty acid substrate [57]. Our homology analysis also identified the amino acid sequence A169-R235, which spans the region of cap-like domain, a subdomain within the α/β hydrolase fold, recently identified in the crystal structure of NDRG2.

The role of PPAS sequence and other domains within NDRG1 is currently unknown. Using mutagenesis and immunofluorescence, we found that expression of Myc-tagged deletion mutants of the HTH and 3xR domains, as well as N- and C- termini (Figure 5A), had no effect on protein localization in hypoxic JEG-3 cells (Figure S2). In contrast, deletion of the entire PPAS domain (residues 128–143) or partial PPAS deletions, PPAS1 (residues 128–133) or PPAS2 (residues 133–143), abrogated nuclear localization of NDRG1 (Figure 5B, and data not shown). A selective deletion of the cap-like domain (residues 169-235), which is also located within the α/β hydrolase, had no effect on the nuclear localization of NDRG1 (Figure 5B). Notably PPAS deletion resulted in reduced protein stability and a faster decay when compared to wild type NDRG1 (Figure S3), which was restored by the proteasome inhibitor MG-132 (Figure 5C, lower panel). However, the restored protein failed to properly localize to the nucleus or membrane-associated structures (Figure 5D).

## Discussion

While NDRG1 is clearly germane to cellular stress response, cancer, and the function of peripheral neurons, the expression pattern and mechanism of action of NDRG1 remain largely unknown. Building upon our previous data on the expression and anti-apoptotic function of NDRG1 in trophoblasts exposed to hypoxia [16,17,48], we showed that the expression of NDRG1 is not upregulated by any cell stressor, but is selectively enhanced by hypoxia or hypoxia mimetic chemicals. Cell stress induced by serum deprivation or UV or ionizing radiation had no effect on the expression of NDRG1. Interestingly, a very prolonged cell culture (7–19 days) without passage, or with several days of cell confluence (not shown), also increased NDRG1 expression as previously reported [23], suggesting that this effect might represent local hypoxia and not nutrient deprivation.

Consistent with its pleiotropic function, NDRG1 has been localized to diverse cellular organelles in different cell types—including the cytoplasm, nucleus, membranes, endosomes, mitochondria, and cytoskeleton [2,33-37]. However, no nuclear localization signal or other membrane anchor motifs have been identified within the protein. By detecting the expression pattern of endogenous or Myc-tagged NDRG1, we found that hypoxia not only upregulated, but also redistributed, trophoblastic NDRG1 to the nucleus as well as to the cytoplasmic membrane. While we identified an HTH motif near NDRG1’s N-terminus, it was unnecessary for nuclear localization.

In contrast, mutation of the PPAS domain abrogated the nuclear redistribution of NDRG1 in hypoxic trophoblasts. The low expression level, perinuclear aggregation, decay, and restoration of cellular levels by MG-132 suggest that PPAS deletion destabilizes NDRG1, shuttling it to proteosomal pathways for degradation. We also noted that the restored NDRG1-ΔPPAS by MG-132 was not properly redistributed as wild type NDRG1 in hypoxic cells. While these data do not prove that the PPAS domain serves as NDRG1’s nuclear localization signal, it is likely that NDRG1-ΔPPAS is disabled secondary to protein misfolding or other structural deformations.

The recently published crystal structure of both human and mouse NDRG2 confirmed the predicted existence of a α/β-hydrolase fold in the Ndr family proteins. This fold consists of two subdomains: a large, canonical α/β-hydrolase domain and a small, cap-like domain. Other than forming a part of the pocket for substrate binding in α/β-hydrolases, the cap-like domain stands out from the main body of α/β-hydrolases, exhibiting multiple surface hydrophobic residues that may contribute to its molecular interactions [51]. The exon-9 skipping mutation of NDRG1, which leads to HSMNL disease, results in amino acid loss from S181-K198 in the cap-like domain, thus highlighting the functional importance of this domain. Our data suggest that the cap-like domain is not necessary for the nuclear localization of NDRG1 in hypoxic trophoblasts.

The function of NDRG1 in the nucleus of hypoxic cells remains unknown. Our data suggest that NDRG1 functions in the nucleus and cytoplasmic membranes during hypoxic stress. Interestingly, a limited number of proteins have been shown to be targets of NDRG1, including proteins associated with tumor growth and metastasis, such as Thtpa, cathepsin C, ATF3 [58], p53 [16,59], E-cadherin [60], the molecular chaperons Hsc70 [61] and Hsp90 [29], and the lipid-related proteins ApoAI and ApoAII [62].

In addition to nuclear translocation, hypoxia also redistributed NDRG1 to the cell membrane and cytoplasmic membranous network closely associated with the ER and microtubules. This suggests an adaptive role for NDRG1 in the biosynthesis and transport of lipids, proteins, and carbohydrates, which are critical functions of placental trophoblasts in supporting the developing embryo. The fact that the PPAS domain is found in ACP proteins of multi-enzyme complexes such as fatty acid synthase [63], together with the observation that NDRG1 binds ApoAI and ApoAII [62], argues that there is a role for NDRG1 in promoting trophoblastic lipid biosynthesis and transport during cell stress.

## Supporting Information

Figure S1 **NDRG1 does not co-localize with several sub-cellular organelles in trophoblastic cells under either standard or hypoxic conditions.** Parental cells or myc-tagged NDRG1 transfectants at 48 h after transfection were incubated in either standard conditions or hypoxia for 24 h. The nuclei (blue) in all panels were detected using Hoechst 33342. Organelle specific antibodies or fluorescent dyes were used to locate subcellular organelles as described in Materials and Methods. (A) Endogenous NDRG1 (green) does not co-localize with mitochondria in JEG-3. (B-C) Myc-tagged NDRG1 (green) does not co-localize with lysosomes (B, red) or Golgi (C, red) in BeWo cells. (D-E) Myc-tagged NDRG1 (red) does not co-localize with peroxisome (D, green) or microfilaments (E, green) in BeWo cells. Data are representative of at least three independent experiments. Bar = 20 µm in all panels.
(TIF)

Figure S2 **The cellular localization of NDRG1 is not affected by N- or C- terminal deleted NDRG1 mutants under hypoxic conditions.** JEG-3 cells were transfected with myc-tagged NDRG1 wild type or deletion mutants at the N- or C- terminal as depicted in Figure 5A. At 48 h after transfection the cells were exposed to CoCl_2_ (200 µM) or vehicle control for 24 h. Myc-tagged NDRG1 (red), E-cadherin (green), and nuclei (blue) were stained as described in Materials and Methods. Data are representative of at least three independent experiments. Bar = 20 µm in all panels.
(TIF)

Figure S3 **Time dependent decay of NDRG1 wild type and PPAS-deleted mutants after inhibition of protein synthesis.** 293T cells were transfected with myc-tagged wild type NDRG1 or PPAS-deleted mutants. 48 h after transfection the cells were exposed to cycloheximide (10 µg/ml) for the time period indicated. NDRG1 protein levels (upper panels) after inhibition of protein synthesis were analyzed by western blot as described in Materials and Methods, with actin (lower panels) as loading control. Data are representative of two independent experiments.
(TIF)
