# Supplementary figures and images for: The Expression and Localization of N-Myc Downstream-Regulated Gene 1 in Human Trophoblasts
Source: PLoS One. 2013 Sep 16;8(9):e75473. doi: 10.1371/journal.pone.0075473 (PMC3774633; doi:10.1371/journal.pone.0075473)

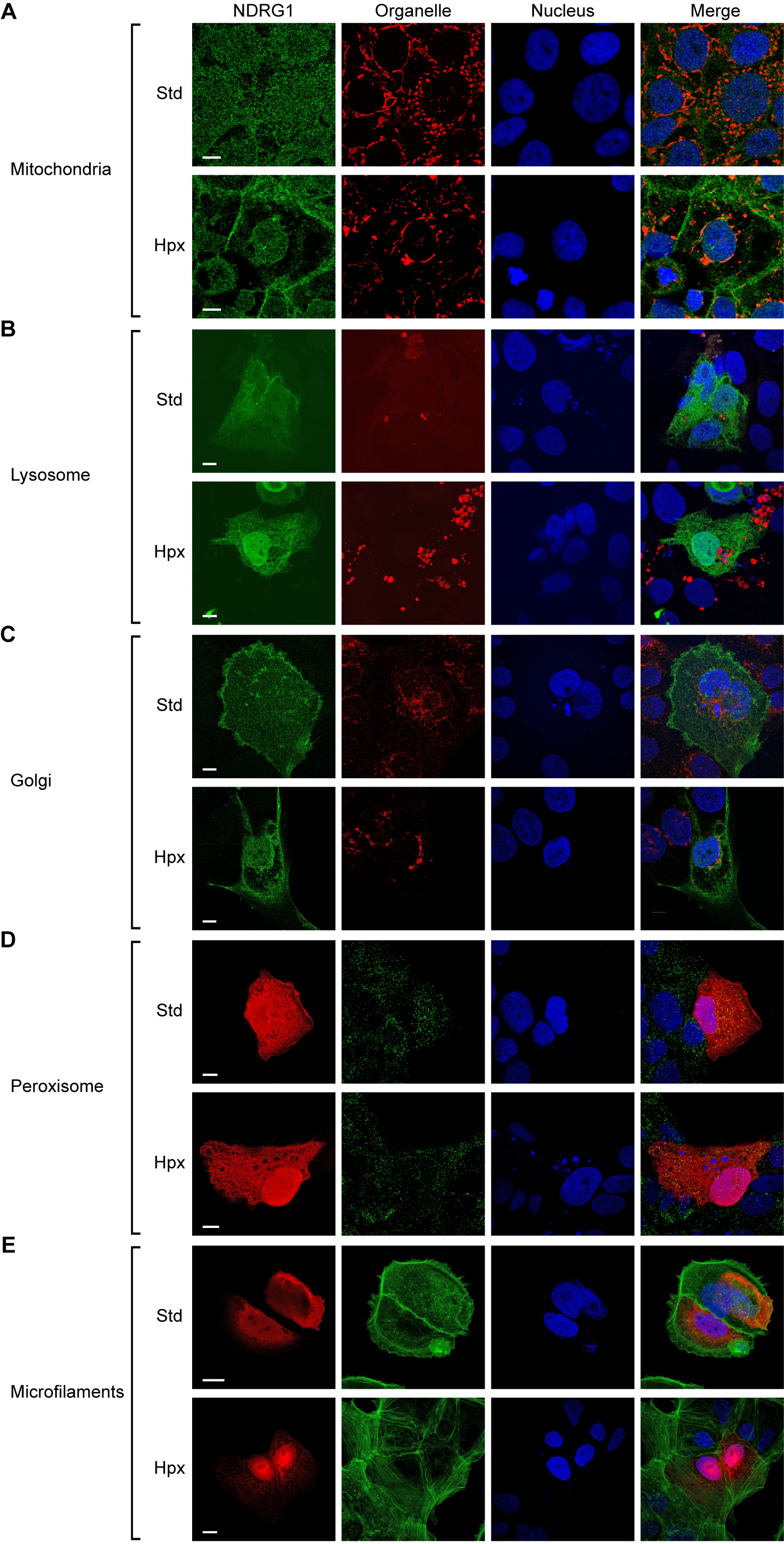

Supplement: Figure S1 — NDRG1 does not co-localize with several sub-cellular organelles in trophoblastic cells under either standard or hypoxic conditions. Parental cells or myc-tagged NDRG1 transfectants at 48 h after transfection were incubated in either standard conditions or hypoxia for 24 h. The nuclei (blue) in all panels were detected using Hoechst 33342. Organelle specific antibodies or fluorescent dyes were used to locate subcellular organelles as described in Materials and Methods. (A) Endogenous NDRG1 (green) does not co-localize with mitochondria in JEG-3. (B-C) Myc-tagged NDRG1 (green) does not co-localize with lysosomes (B, red) or Golgi (C, red) in BeWo cells. (D-E) Myc-tagged NDRG1 (red) does not co-localize with peroxisome (D, green) or microfilaments (E, green) in BeWo cells. Data are representative of at least three independent experiments. Bar = 20 µm in all panels. (TIF) [file pone.0075473.s001.tif]

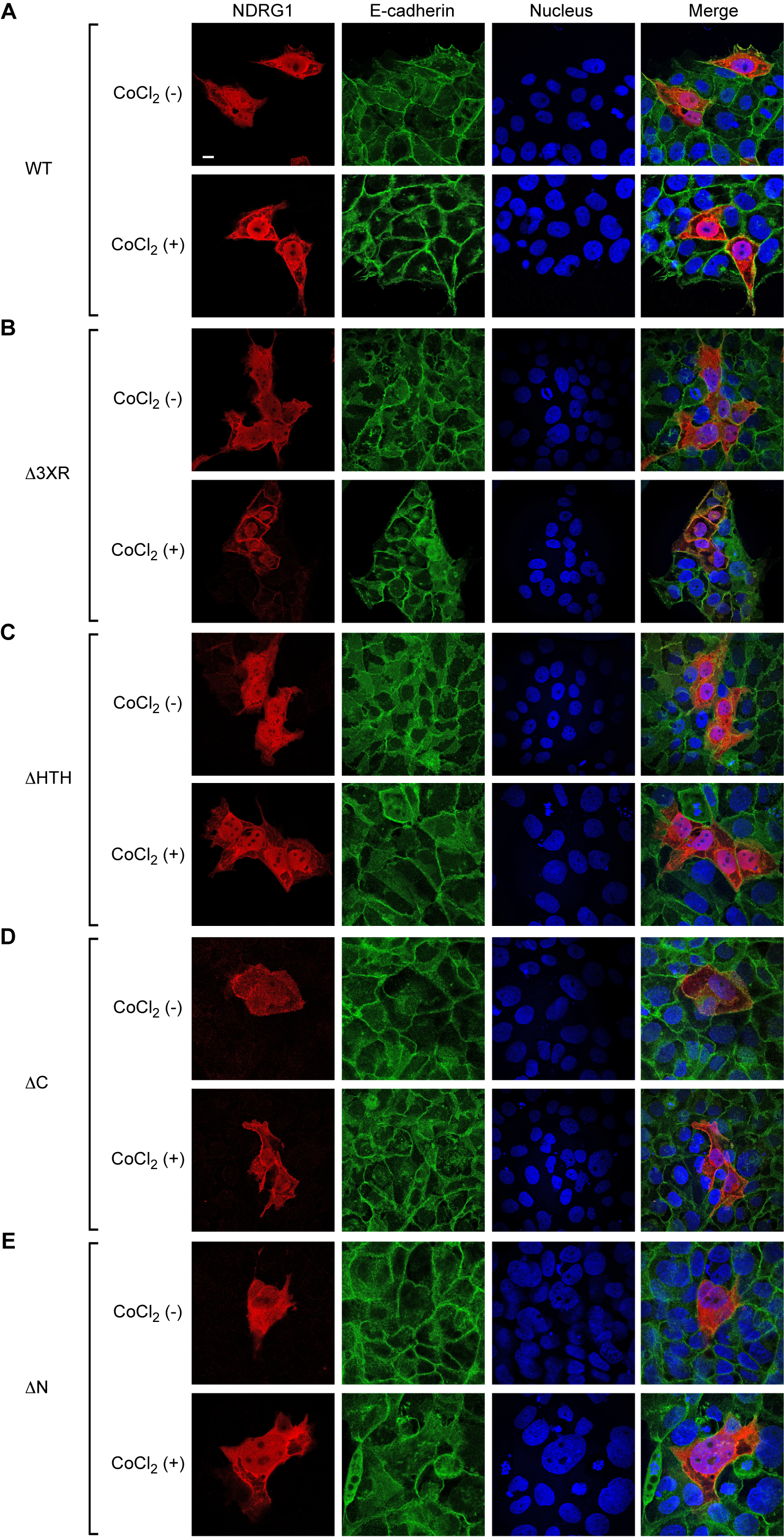

Supplement: Figure S2 — The cellular localization of NDRG1 is not affected by N- or C- terminal deleted NDRG1 mutants under hypoxic conditions. JEG-3 cells were transfected with myc-tagged NDRG1 wild type or deletion mutants at the N- or C- terminal as depicted in Figure 5A. At 48 h after transfection the cells were exposed to CoCl2 (200 µM) or vehicle control for 24 h. Myc-tagged NDRG1 (red), E-cadherin (green), and nuclei (blue) were stained as described in Materials and Methods. Data are representative of at least three independent experiments. Bar = 20 µm in all panels. (TIF) [file pone.0075473.s002.tif]

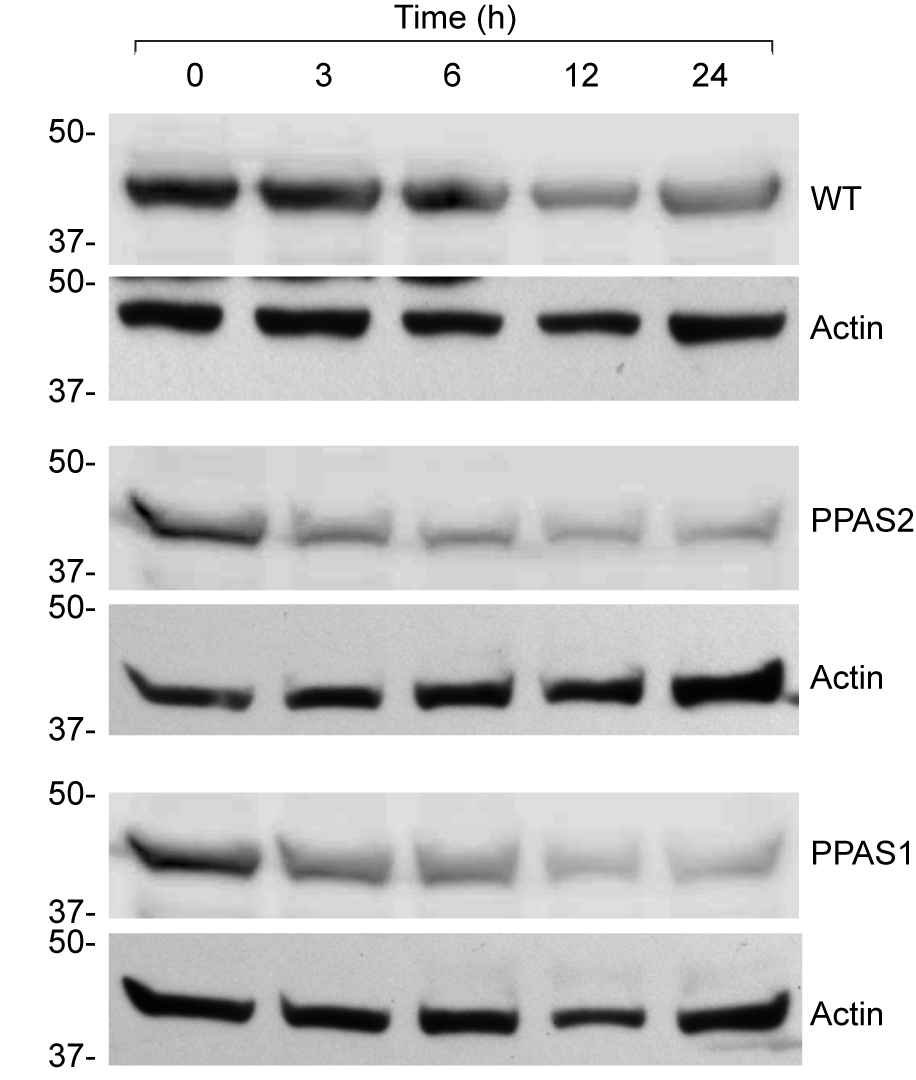

Supplement: Figure S3 — Time dependent decay of NDRG1 wild type and PPAS-deleted mutants after inhibition of protein synthesis. 293T cells were transfected with myc-tagged wild type NDRG1 or PPAS-deleted mutants. 48 h after transfection the cells were exposed to cycloheximide (10 µg/ml) for the time period indicated. NDRG1 protein levels (upper panels) after inhibition of protein synthesis were analyzed by western blot as described in Materials and Methods, with actin (lower panels) as loading control. Data are representative of two independent experiments. (TIF) [file pone.0075473.s003.tif]
